# Deodorants for Iodoform

**Published:** 1888-06

**Authors:** 


### Deodorants for Iodoform.

Oil of turpentine has been recommended as a very efficient deodorant for the hands or for utensils, after they have come in contact with iodoform. They are first thoroughly rubbed with oil of turpentine, and, after about half a minute, washed with soap or soap spirit. It is also asserted that a watery solution of tannic acid, which leaves no odor of its own, destroys that of iodoform immediately, and can be washed off at once.

